# New Strategy for Rapid Diagnosis and Characterization of Fungal Infections: The Example of Corneal Scrapings

**DOI:** 10.1371/journal.pone.0037660

**Published:** 2012-07-02

**Authors:** Pablo Goldschmidt, Sandrine Degorge, Patricia Che Sarria, Djida Benallaoua, Oudy Semoun, Vincent Borderie, Laurent Laroche, Christine Chaumeil

**Affiliations:** 1 Centre Hospitalier National d’Ophtalmologie des Quinze-Vingts, Paris, France; 2 Laboratoire Jean Dausset, Hôpital Saint Louis, Paris, France; University of Houston, United States of America

## Abstract

**Purpose:**

The prognosis of people infected with *Fungi* especially immunocompromised depends on rapid and accurate diagnosis to capitalize on time administration of specific treatments. However, cultures produce false negative results and nucleic-acid amplification techniques require complex post-amplification procedures to differentiate relevant fungal types. The objective of this work was to develop a new diagnostic strategy based on real-time polymerase-chain reaction high-resolution melting analysis (PCR-HRM) that a) detects yeasts and filamentous *Fungi*, b) differentiates yeasts from filamentous *Fungi*, and c) discriminates among relevant species of yeasts.

**Methods:**

PCR-HRM detection limits and specificity were assessed with a) isolated strains; b) human blood samples experimentally infected with *Fungi*; c) blood experimentally infected with other infectious agents; d) corneal scrapings from patients with suspected fungal keratitis (culture positive and negative) and e) scrapings from patients with suspected bacterial, viral or *Acanthamoeba* infections. The DNAs were extracted and mixed with primers diluted in the MeltDoctor® HRM Master Mix in 2 tubes, the first for yeasts, containing the forward primer CandUn (5'CATGCCTGTTTGAGCGTC) and the reverse primer FungUn (5'TCCTCCGCTT ATTGATATGCT) and the second for filamentous *Fungi*, containing the forward primer FilamUn (5'TGCCTGTCCGAGCGTCAT) and FungUn. Molecular probes were not necessary. The yields of DNA extraction and the PCR inhibitors were systematically monitored.

**Results:**

PCR-HRM detected 0.1 Colony Forming Units (CFU)/µl of yeasts and filamentous *Fungi*, differentiated filamentous *Fungi* from yeasts and discriminated among relevant species of yeasts. PCR-HRM performances were higher than haemoculture and sensitivity and specificity was 100% for culture positive samples, detecting and characterizing *Fungi* in 7 out 10 culture negative suspected fungal keratitis.

**Conclusions:**

PCR-HRM appears as a new, sensitive, specific and inexpensive test that detects *Fungi* and differentiates filamentous *Fungi* from yeasts. It allows direct fungal detection from clinical samples and experimentally infected blood in less than 2.30 h after DNA extraction.

## Introduction

The frequency of fungal infections has been increasing for the last 30 years due to viral or iatrogenic immunodeficiencies, the efficiency in treating bacterial infections, the development of in-dwelling devices, and the massive use of contact lenses.[1]–[8] The incidence of fungal keratitis (keratomycosis) is also on the rise, and filamentous *Fungi* are the most frequently reported pathogens. [3], [8] From yeasts, *Candida albicans* is the most frequently associated with disease. However, *C. glabrata, C. tropicalis, C. krusei, and C. parapsilosis* have gained greater significance. [1], [2], [4], [8].

Fungal infection management requires timely diagnosis for rapid onset of treatments, but approximately one half of the samples remain culture negative and/or negative by fungal antigen detection using immunosorbent assays (ELISA).[9]–[10] Improved detection performances were reported by amplifying fungal genomic regions (polymerase chain reactions, PCRs). [11], [12] However, the classic PCRs do not differentiate filamentous *Fungi* from yeasts and require post amplification procedures (restriction enzyme digestion and analysis; single-base extension; hybridization probes or molecular sequencing).[11]–[15] The “gold standard” for fungal characterization is DNA sequencing, but this method is laborious, expensive and cannot be performed routinely for daily diagnosis. [15].

The real-time Taqman PCR using fluorogenic labelled Taqman-probes facilitates the detection and partial characterization of *Fungi* but requires a series of expensive labelled probes (each probe detects a single fungal type or one species per reaction). [12], [16]–[17].

Because the first-line therapy is different for filamentous *Fungi* and yeasts as well as for different yeasts, rapid and accurate information is required to target the treatments according to natural fungal susceptibilities.[18]–[20].

The availability of improved fluorescent DNA binding dyes with highly predictable saturation properties allows precise assessment of sequence length by High Resolution Melting real-time PCR (PCR-HRM). [21], [22] Recently, a diagnosis test based on PCR-HRM technology was reported for vaginal samples, detecting and identifying 8 *Candida* at species level. [23] Nevertheless, it was unable to differentiate *Candida* from filamentous *Fungi* and did not detect and characterise *S. cervisiae* and *Trichosporon*.

The goal of the present work is to develop a new test able to detect in 1 run the equivalent of at least 1 fungal colony forming unit (CFU) per reaction. In addition this molecular approach should simultaneously differentiate yeasts from filamentous *Fungi* and discriminate among relevant species of yeasts in clinical samples and in blood experimentally infected with fungal suspensions.

## Materials and Methods

Investigations were conducted according to the principles expressed in the Declaration of Helsinki (http://www.wma.net/e/policy/) and were approved by the Institutional Review Board of the Centre Hospitalier National des Quinze-Vingts (CHNO), Ministry of Public Health, Paris-France. Written informed consent was obtained from all participants for the use of each sample. Forms with written consent were drafted according to the requirements of the CHNO Review Board and the National Health Authorities were double checked, validated and signed by the physician in charge of the sampling and sent to the laboratory. The preliminary studies were performed with characterized strains isolated from patients presenting corneal ulcers in the National Eye Hospital in Paris (CHNO des Quinze-Vingts) or from strains isolated from blood stream infections (generous gift from Dr Christophe Hennequin’s laboratory, CHU Saint-Antoine, Paris, France).

**Table 1:** High-resolution melting analysis (PCR-HRM) detection limits (fungal spore suspensions titrated by plating) and discrimination among fungal species using the primers CandUn + FungUn and FilamUn + FungUn.

			Set of primers			
			CandUn* + FungUn**		FilamUn° + FungUn**	
			HRM detectionlimit (CFU/µl)	Differential profiles	HRM detection limit (CFU/µl)	Differential profiles
				I from II I from III II from IIIDifferential profiles for Ia Ib; Ic; Id; Ie;If and Ig HRMProfiles were similarfor II h; II i; II j and II k		I from II I from III II from III
**Ref**		**Strain**				
I	a	*Candida tropicalis*	≤0.1		≥1	
	b	*Candida parapsilosis*	≤0.1		≥1	
	c	*Candida albicans*	≤0.1		≥1	
	d	*Candida glabrata*	≤0.1		≥1	
	e	*Candida krusei*	≤0.1		≥1	
	f	*Saccharomyces cervisiae*	≤0.1		≥1	
	g	*Trichosporon*	≤0.1		≥1	
II	h	*Aspergillus nidulans*	≥5		≤0.1	
	i	*Aspergillus niger*	≥5		≤0.1	
	j	*Penicillium piccum*	≥5		≤0.1	
	k	*Aspergillus sp.*	≥5		≤0.1	
III	l	*Fusarium solani*	≥5		≤0.1	

I: yeast; II and III: Filamentous *Fungi*;

* CandUn sequence: 5' CATGCCTGTTTGAGCGTC;

°FilamUn sequence: 5' TGCCTGTCCGAGCGTCAT;

** FungUn sequence: 5' TCCTCCGCTTATTGATATGCT.

**Figure 1:**
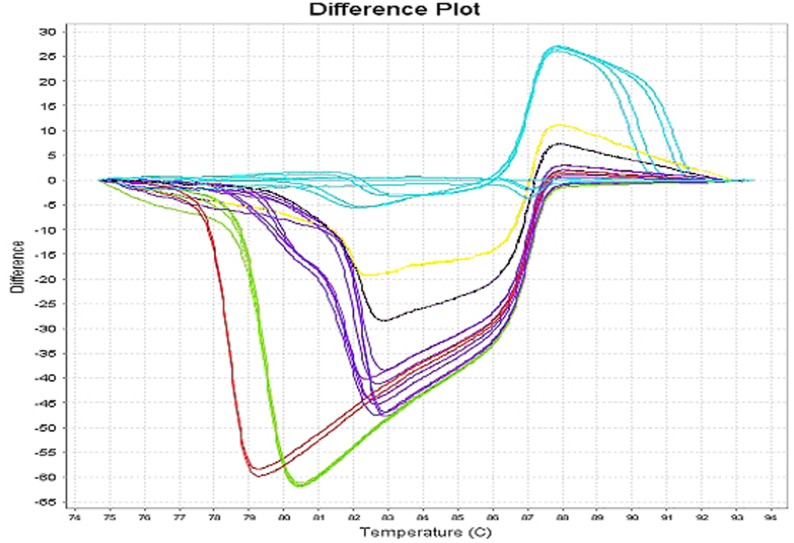
PCR-HRM profiles obtained with yeasts and filamentous *Fungi* using the primers CandUn + FungUn. In red: *Candida tropicalis*; green: *C. parapsilopsis*; violet: *C. albicans*; black: *C. glabrata*; yellow: *C. krusei*; blue: Filamentous *Fungi* (*Aspergillus fumigatus* and *Fusarium solani*.).

**Figure 2:**
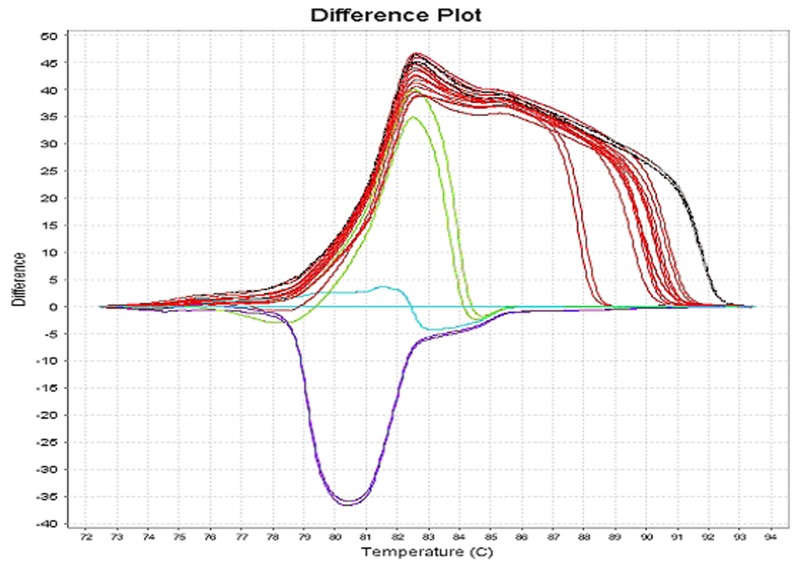
PCR-HRM profiles obtained with yeasts and filamentous *Fungi* using the primers FilamUn + FungUn. In red: *Aspergillus sp*.; black: *Fusarium solani*; violet, blue and green: *Candida sp*.

One colony of each fungal species was scraped from the surface after 48 h of culture on Sabouraud’s dextrose agar, suspended in Phosphate Buffer Solution (PBS) and replated. To reduce the over representation of fungal DNA from non viable organisms, one colony was scraped from the second dish 48 hours later, suspended in PBS and tenfold diluted. Each dilution was divided in several aliquots; three were plated on Sabouraud’s dextrose agar to assess the number of colonies (equivalent CFU/ml) and the others kept as calibrators. For each series of experiments the PCR-HRM detection limits were validated with serial dilutions of fungal suspensions diluted in PBS and simultaneously titrated by plating.

Blood samples were collected in 10 ml citrate tubes from vein puncture of healthy subjects and transported to the laboratory within 1 h. After white cell count to assess the cell load of inoculums (white cell counts >12.000/µl were excluded) randomized aliquots were spiked with different titrated fungal suspensions. Negative controls consisted in non infected blood or leukocyte suspensions from the same individuals.

**Table 2:** Comparison of direct microscopic examination, culture and high-resolution melting analysis performances (PCR-HRM) on corneal scrapings obtained from patients with keratitis.

Sample Number	Diagnostic method			
	microscopic examination* #	Culture	PCR (*Herpes simplex and Acanthamoeba*)	HRM results and profiles
1	yeasts	*Candida glabrata*	NEG	*Candida glabrata*
2	GNR	*Pseudomonas aeruginosa*	NEG	NEG
3	budding yeasts	*Candida albicans*	NEG	*Candida albicans*
4	GPC	*Staphylococcus aureus*	NEG	NEG
5	yeasts	*Candida krusei*	NEG	*Candida krusei*
6	Yeasts; pseudohypha	*Saccharomyces cervisiae*	NEG	*S. cervisiae*
7	GPR	*Corynebacteria*	NEG	NEG
8	filaments	*Aspergillus nidulans*	NEG	Filamentous *Fungi*
9	LY; AEC	NEG	POS HSV1	NEG
10	filaments	*Penicillium piccum*	NEG	Filamentous *Fungi*
11	LY; AEC	NEG	POS HSV2	NEG
12	NEG	*Candida tropicalis*	NEG	*Candida tropicalis*
13	filaments	*Fusarium solani*	NEG	*Fusarium sp.*
14	GPC	*Staphylococcus epidermidis*	NEG	NEG
15	GNR	*Escherichia coli*	NEG	NEG
16	GNR	*Acinetobacter baumannii*	NEG	NEG
17	yeasts	*Trichosporon*	NEG	POS; Trichosporon
18	yeasts	*Candida parapsilosis*	NEG	*Candida parapsilosis*
19	GPC	*Streptococcus agalactiae*	NEG	NEG
20	FF	*Aspergillus niger*	NEG	Filamentous *Fungi*
21	GNR	*Pseudomonas aeruginosa*	NEG	NEG
22	AEC	NEG	POS AC	NEG
23	NEG	*Aspergillus niger*	NEG	Filamentous *Fungi*
24	filaments	*Fusarium solani*	NEG	*Fusarium sp.*
25	GPR	*Propionibacterium acnes*	NEG	NEG
26	GPC	*Staphylococcus epidermidis*	NEG	NEG
27	GNR	*Pseudomonas aeruginosa*	NEG	NEG
28	GPC	*Streptococcus pneumoniae*	NEG	NEG
29	filaments	NEG	NEG	Filamentous *Fungi*
30	NEG	NEG	NEG	NEG
31	filaments	NEG	NEG	*Fusarium sp.*
32	budding yeasts	NEG	NEG	*Candida albicans*
33	NEG	NEG	NEG	NEG
34	NEG	NEG	NEG	Filamentous *Fungi*
35	NEG	NEG	NEG	NEG
36	filaments	NEG	NEG	Filamentous *Fungi*
37	NEG	NEG	NEG	Filamentous *Fungi*
38	NEG	NEG	NEG	*Candida albicans*
**Controls ##**				
39	NEG	10^6^ Human epithelial cells	NEG	NEG
40	NEG	Distilled water	NEG	NEG
41	NEG	Saline	NEG	NEG
42	NEG	DNA extraction reactants	NEG	NEG
43	NEG	10^6^ Human fibroblasts	NEG	NEG
44	NEG	Transport media	NEG	NEG
45	NEG	Laminar-flow air control	NEG	NEG
46	NEG	10^6^ Human leukocytes **	NEG	NEG
47	NEG	Whole blood donor 1	NEG	NEG
48	NEG	Whole blood donor 2	NEG	NEG
49	NEG	Whole blood donor 3	NEG	NEG
50	NEG	Whole blood donor 4	NEG	NEG
51	NEG	10^6^ Human leukocytes ^a^	NEG	NEG
52	NEG	10^7^ Human leukocytes ^a^	NEG	NEG
53	NEG	10^8^ Human leukocytes ^a^	NEG	NEG
54	NEG	10^9^ Human leukocytes ^a^	NEG	NEG
55	NEG	Air	NEG	NEG
56	NEG	Laminar flow (air)	NEG	NEG
57	NEG	Laminar flow (surface)	NEG	NEG
58	NEG	DNA extraction reactants	NEG	NEG
59	NEG	Laminar flow (surface)	NEG	NEG
60	NEG	Air (laboratory)	NEG	NEG
61	NEG	Laminar flow (air)	NEG	NEG
62	NEG	DNA extraction reactants	NEG	NEG

HRM: high-resolution melting analysis; *: after Giemsa (pH: 7.4) and Grocott staining; POS: Positive; NEG: Negative**;** FF: Filamentous Fungi**;** GNR: Gram-negative rods; GPR: Gram-positive rods; GPC: Gram-positive *cocci*; LY: lymphocytes; AEC: Altered epithelial cells; HSV1: *Herpes-simplex* virus type 1; HSV2: *Herpes-simplex* virus type 2; AC: *Acanthamoeba*; **: DNA was extracted from 6 different individuals and tested separately; ##: controls were extracted and tested for each run; ^a^: The leukocytes from 4 different donors were extracted and tested separately. Air and working surface samples were obtained placing open tubes for 15 minutes under the laminar flow or in the laboratory working table. Surface samples were collected with cotton devices humidified with saline by gentle swabbing the working surface. DNA extraction procedures were conducted in duplicate in each series of experiments for the DNA extraction reactants.

For each fungal strain, haemoculture bottles were inoculated with 10 ml of saline or blood spiked or not with *Fungi* and incubated up to 12 days before discarded. Fungal isolates were phenotypically characterized by conventional tests [Chromagar *Candida* BR, ref 257480 (Becton Dickinson, France); API 20AUX ref. 20210 (Biomérieux, France); API Candida ref 10500 (Biomérieux, France) and Lactophenol Blue, ref 363060-0125, RAL Advanced Chromatic, France)]. The confirmation of fungal species was carried out by sequencing the DNA from both ends using flanking vector primers. [12], [15] Bacteria were cultured and characterized with routine diagnosis tests; *Acanthamoeba* and *Herpesviridae* were detected by real-time PCR. [24], [25].

**Table 3:** Comparison of culture and high-resolution melting analysis (PCR-HRM) performances on experimentally infected blood.

	Fungal load [a]CFU/ml	Fluid	Diagnosis method						
			Fungal Culture #				HRM #		
			Result		Hours for positivity		Result		Profile ##
*Candida albicans*	10	S	POS	POS	16	16	POS	POS	*C. albicans*
		B	POS	POS	24	24	POS	POS	*C. albicans*
	1	S	POS	POS	48	24	POS	POS	*C. albicans*
		B	POS	POS	48	48	POS	POS	*C. albicans*
	0.1	S	POS	NEG	96	–	POS	POS	*C. albicans*
		B	NEG	NEG	–	–	POS	POS	*C. albicans*
*Candida krusei*	10	S	POS	POS	24	24	POS	POS	*C. krusei*
		B	POS	POS	24	24	POS	POS	*C. krusei*
	1	S	POS	POS	48	48	POS	POS	*C. krusei*
		B	POS	POS	72	48	POS	POS	*C. krusei*
	0.1	S	POS	NEG	96	–	POS	POS	*C. krusei*
		B	NEG	NEG	–	–	POS	NEG	*C.krusei*
*Candida glabrata*	10	S	POS	POS	24	24	POS	POS	*C. glabrata*
		B	POS	POS	24	24	POS	POS	*C. glabrata*
	1	S	POS	POS	48	36	POS	POS	*C. glabrata*
		B	POS	POS	48	72	POS	POS	*C. glabrata*
	0.1	S	NEG	NEG	–	–	POS	POS	*C. glabrata*
		B	NEG	NEG	–	–	POS	POS	*C. glabrata*
*Candida tropicalis*	10	S	POS	POS	36	36	POS	POS	*C. tropicalis*
		B	POS	POS	48	48	POS	POS	*C. tropicalis*
	1	S	POS	POS	48	48	POS	POS	*C. tropicalis*
		B	POS	POS	48	72	POS	POS	*C. tropicalis*
	0.1	S	NEG	POS	–	96	POS	POS	*C. tropicalis*
		B	NEG	NEG	–	–	POS	POS	*C. tropicalis*
*Candida parapsilopsis*	10	S	POS	POS	24	24	POS	POS	*C. parapsilopsis*
		B	POS	POS	24	24	POS	POS	*C. parapsilopsis*
	1	S	POS	NEG	36	48	POS	POS	*C. parapsilopsis*
		B	NEG	POS	48	36	POS	POS	*C. parapsilopsis*
	0.1	S	NEG	NEG	–	–	POS	POS	*C. parapsilopsis*
		B	NEG	NEG	–	–	POS	POS	*C. parapsilopsis*
*Saccharomyces cervisiae*	10	S	POS	POS	24	24	POS	POS	*S. cervisiae*
		B	POS	POS	24	24	POS	POS	*S. cervisiae*
	1	S	POS	POS	24	24	POS	POS	*S. cervisiae*
		B	POS	POS	36	36	POS	POS	*S. cervisiae*
	0.1	S	NEG	NEG	–	–	POS	POS	*S. cervisiae*
		B	NEG	NEG	–	–	POS	POS	*S. cervisiae*
*Trichosporon*	10	S	POS	POS	24	36	POS	POS	*Trichosporon*
		B	POS	POS	36	36	POS	POS	*Trichosporon*
	1	S	POS	POS	48	36	POS	POS	*Trichosporon*
		B	NEG	POS	48	48	POS	POS	*Trichosporon*
	0.1	S	POS	NEG	96	–	POS	POS	*Trichosporon*
		B	NEG	NEG	–	–	POS	POS	*Trichosporon*
*Aspergillus niger*	10	S	POS	POS	36	48	POS	POS	*Filamentous Fungi*
		B	POS	POS	48	48	POS	POS	*Filamentous Fungi*
	1	S	POS	POS	48	72	POS	POS	*Filamentous Fungi*
		B	POS	NEG	72	96	POS	POS	*Filamentous Fungi*
	0.1	S	NEG	NEG	–	–	POS	POS	*Filamentous Fungi*
		B	NEG	NEG	–	–	POS	POS	*Filamentous Fungi*
*Aspergillus nidulans*	10	S	POS	POS	48	48	POS	POS	*Filamentous Fungi*
		B	POS	POS	48	72	POS	POS	*Filamentous Fungi*
	1	S	POS	POS	48	48	POS	POS	*Filamentous Fungi*
		B	POS	POS	72	72	POS	POS	*Filamentous Fungi*
	0.1	S	POS	NEG	–	–	POS	POS	*Filamentous Fungi*
		B	NEG	NEG	–	–	POS	POS	*Filamentous Fungi*
*Penicillium piccum*	10	S	POS	POS	24	36	POS	POS	*Filamentous Fungi*
		B	POS	POS	36	36	POS	POS	*Filamentous Fungi*
	1	S	POS	POS	72	72	POS	POS	*Filamentous Fungi*
		B	POS	POS	72	96	POS	POS	*Filamentous Fungi*
	0.1	S	POS	NEG	–	–	POS	POS	*Filamentous Fungi*
		B	NEG	NEG	–	–	POS	POS	*Filamentous Fungi*
*Fusarium solani*	10	S	POS	POS	36	48	POS	POS	* Filamentous Fungi
		B	POS	POS	48	48	POS	POS	* Filamentous Fungi
	1	S	POS	POS	48	72	POS	POS	* Filamentous Fungi
		B	POS	POS	72	48	POS	POS	* Filamentous Fungi
	0.1	S	NEG	NEG	–	–	POS	POS	* Filamentous Fungi
		B	NEG	NEG	–	–	POS	POS	* Filamentous Fungi

[a] Haemoculture bottles were inoculated with 10 ml; S: saline; B: blood; #: results from 2 independent experiments; POS: Positive; NEG: Negative after 240 hours**;** ##: reference strains and negative controls were extracted and tested for each run;*: melting-curve profiles consistently superimposed on those obtained with *Fusarium solani*.

Corneal scrapings from 38 patients, 13 with proven fungal culture positive, 10 with suspected fungal keratitis culture negative and 15 with non suspected fungal keratitis (bacterial, viral or *Acanthamoeba*) were tested masked. Sampling from patients presenting corneal ulcers and requiring microbiological diagnosis was performed by deep corneal scraping by certified ophthalmologists with sterile stainless steel blades after rinsing of fluorescein and topical anaesthetic from the eye surface. [24] Slides with aliquots of scrapings were fixed and stained (Giemsa pH: 7.4) for direct microscopic examination and the presence of *Fungi* was confirmed by Grocott's methenamine silver reaction. The second aliquots were cultured within 30 minutes after collection up to 30 days before discarded as culture negative and remnants of blades were frozen dry at –80°C for further molecular diagnosis. PCR-HRM was carried out after thawing and addition of 200 µl of sterile Phosphate Buffer Solution (PBS) to the tubes containing the dry blades.

The DNA extraction was carried out in a vertical safety laminar flow cabinet in a dedicated room. To monitor the extraction yields and the absence of PCR inhibitors the internal control (IC) consisting of 5 µl of a whole virus preparation of seal herpes virus (gift from G. J. van Doornum, Dept. of Virology Erasmus MC, Rotterdam, The Netherlands) was added to 200 µl of each suspension (scraping, blood, leukocytes or saline) before extraction (final concentration of 1000 to 2000 viral particles/ml). [24], [25] In order to obtain spheroplasts, each specimen (sample + IC) was mixed with tris-EDTA buffer and 10 U recombinant lyticase (Sigma-Aldrich, France)/100 µl of suspension and incubated at 37°C for 60 min. After incubation, the suspensions were vortexed thoroughly and 100 µl were used for DNA extraction using the MagNA Pure compact nucleic acid isolation kit I® as described by the manufacturer in the MagNA Pure Compact automate® (Roche Diagnostics, Meylan, France) and eluted in 100 µl of elution buffer. To monitor the DNA extraction yields and the PCR inhibitors the seal herpes virus internal control (IC) was amplified in an independent real-time PCR run. [24], [25] The primer sequences were respectively: 5′GGGCGAATCACAGATTGA ATC and 5′GCGGTTCCAAACGTACCAA and VIC-TTTTTATGTGTCCGCCACCATCT GGATC-TAMRA for the probe. Amplification and detection of the IC was carried out in a separate tube containing 18.5 µl of the TaqMan® FAST Universal PCR Mastermix (2X no Amperase® UNG) (Applied Biosystems-France ABI Ref. 4352042), the forward and the reverse primers (0.5 uM each) with or without the fluorophore-labelled TaqMan® probe (0.5 uM). This solution was mixed with 5 µl of the DNA eluted in DNA and RNA-free solution. The PCR cycling program consisted of one cycle at 95°C for 20 sec and 45 cycles at 95°C for 3 sec and 30 sec at 60°C. [24].

Kits (stable at −20°C for at least 12 weeks) for fungal detection consist of 2 tubes, the first for detection, semi quantification and identification of yeasts; the second for detection and semi quantification of filamentous *Fungi*. Each tube contains 10 µl of MeltDoctor® HRM Master Mix (MDHRM) (ABI Applied Biosystems-France Ref 4415440), and 1 µL of the forward and 1 µl of the reverse primer, each at 300 nM (final concentration).

For the detection, quantification and characterization of yeasts and Filamentous *Fungi* the primers were selected in a region bracketing significant polymorphisms of multicopy ribosomal genes of the 18 S ribosomal RNA gene. The Primer 1: HRM CandUn1∶5′CATGCCTGTTTGAGCGTC (conserved sequences of yeasts,) and the Primer 2: HRM FungUn: 5′TCCTCCGCTTATTGATATGCT (conserved regions of all *Fungi*) allow obtaining profiles for the different yeasts according to the sizes of amplicons (alignment of sequences according to EMBL data library). The amplicon sizes (nucleotides bracketed by the primers CandUn + FungUn) are 189 for *Candida albicans,* 192 for *C. dubliniensis* (isolate M334a), 270 for *C. Glabrata,* 199 for *Issatchenkia orientalis (C. krusei*), 269 for *C. nivariensis* (isolate VPCI 1293), 166 for *C. metapsilosis* (strain CBS-2916), 162 for *C. parapsilopsis,* 179 for *C. tropicalis* and 225 for *C. zeylanoides* (strain TJY13a 2).

For filamentous *Fungi* the selected sequences for HRM are FilamUn: 5′TGCCTGTTCCGAGCGTCAT (forward primer) and HRM FungUn: 5′TCCTCCGCTTAT TGATATGCT. The amplicons sizes are 189 for *A. versicolor* and *A. heteromorphus,* 190 for *A. sidowi; A. carbonarius* and *Aspergillus sp.,* 191 for *A. oryzae* and *A. niger*, and 192 for *A. brasiliensis; A. flavus; A. toxicariu* and *A. bombycis*. For *Fusarium napiforme* and for *F. solani* (isolates FMR 799; FMR 4389; 4391; FMR 7338-42; FMR 7991; FMR 7993; FMR 7989; 7994; FMR isolates 7995 to 8000 and FMR 8013) the amplicons are sizes are ranged between 194 and 196 nucleotides. For *F. polyphialidicum*; *F. redolens*; *F. proliferatum; F. proliferatum*; *F. beomiforme* and *F. fujikuroi* amplicon sizes are 206, and for *F. proliferatum*; *F. fujikuroi*; *F. dlaminii* 201 nucleotides.

For PCR-HRM the DNA extracts (10 µl) were introduced in 2 tubes, the first containing CandUn + FungUn in the MDHRM the second FilamUn + FungUn. During PCR-HRM, the amplicons were automatically measured in a closed tube format using integrated cycler/fluorimeter ABI 7500 upgraded equipment and monitored using fluorescent DNA intercalating dyes present in the MDHRM. The PCR program started with a denaturation of 10 min at 95°C, followed by 55 cycles of amplification (15 s at 95°C, 30 s at 60°C and 30 s at 72°C). The PCR-HRM curve was obtained by denaturation at 95°C for 15 sec, cooling to 50°C for 1 min and a temperature increase until 60°C for 15 sec with a 2.2°C/s ramp rate. Samples with fluorescence of less than the 100% of the maximum were excluded from the analysis. Each run contained negative controls with no template and DNA extracts from the reactants. Linearity, sensitivity and detection limit (Equivalent CFU/ml from the Ct versus dilution curves) and reproducibility were assessed by diluting fungal suspensions in distilled water before DNA extraction. The melting temperature (Tm) at which 50% of the DNA is in the double stranded state was assessed by taking the derivative of the melting curve. The melting curves shapes depend on PCR product (amplicon) length. The DNA patterns of the derivative plot (difference plot) were used for amplicon analysis.

## Results

Preliminary experiments were performed to assess the best conditions for extraction of DNA from spores: a- heat for 10 min at 94°C; b- proteinase K at 37°C for 60 min and heat at 94°C for 10 min; c- proteinase K at 37°C for 60 min, heat at 94°C for 10 min and extraction with the MagNA Pure compact nucleic acid isolation kit I® as described by the manufacturer in the MagNA Pure Compact® automate (Roche Diagnostics, Meylan, France); d- shaked in presence of beads and extraction with MagNA Pure; e- shaked in presence of beads with or without proteinase K at 37°C for 60 min, heat at 94°C for 10 min and extraction with Magna Pure; f- shaked in presence of beads with or without lyticase at 37°C for 60 min and heat at 94°C for 10 min; g- shaked in presence of beads with lyticase at 37°C for 60 min, heat at 94°C for 10 min and extracted with Magna Pure; or h- lyticase at 37°C for 60 min, heat at 94°C for 10 min and extraction with Magna Pure. The highest fungal DNA extraction rates were obtained using the procedures g or h (results not shown).

The detection limits have been obtained by dilution of fresh titrated fungal suspensions. PCR-HRM with the primers CandUn + FungUn detected 0.1 CFU/µl of *Candida albicans, C. krusei, C. glabrata, C. tropicalis, Saccharomyces cervisiae* and *Trichosporon* and 1 CFU/ml of filamentous *Fungi* suspended in PBS. As shown in Table 1 PCR-HRM detection capacities were repeatedly higher for yeasts (10 to 100 times) using the set CandUn + FungUn, and more than 10 times higher for filamentous *Fungi* using the set FilamUn + FungUn (*Aspergillus nidulans, A. niger, A. versicolor, A. terreus, Penicillium piccum and Fusarium solani*). These results suggest that the optimization of fungal detection requires the simultaneous amplification of DNA extracts in 2 tubes, the first with the set CandUn + FungUn and the second with FilamUn + FungUn. Under these conditions, the PCR-HRM coefficient of variation for the interassay reproducibility in the complete linear range of detection [10^5^ to 10^−1^ colony forming units (CFU)/ml] for 5 runs was less than 10%. The patterns of the first derivative (difference plot) permitted differentiation of yeasts from filamentous *Fungi* and the divergence between amplicon sizes of closely related species allowed PCR-HRM to easily discriminate among yeasts (Figure 1). According to the amplicon sizes bracketed by the set of primers FilamUn + FungUn (generally ≥194 nucleotides for *Fusarium sp*. versus ≤192 for most species of *Aspergillus*) the melting curve shapes were repeatedly different for *Fusarium solani* (Figure 2).

Sensitivity and specificity of PCR-HRM (while comparing with corneal scraping cultures) was of 100%. PCR-HRM allowed rapid diagnosis of keratomycosis differentiating clinical relevant species of yeasts, and produced negative results for all the samples obtained from patients with non suspected fungal keratitis and for all the negative controls [DNA extracted from 10^6^ CFU/ml of *Bacteria*, 10^6^ PFU (plaque forming units)/ml of *Herpes simplex virus* type 1 or 10^6^ PFU/ml of *Herpes simplex virus* type 2, and 10^5^ *Acanthamoeba* cysts/ml suspended in saline] (Table 2). Human cells (10^6^ human epithelial cells or fibroblasts) did not interfere with the PCR-HRM performances, confirming the *in-silico* specificity predictions. In patients with clinically suspected fungal keratitis (samples 1; 3; 5; 6; 8; 10; 12; 13; 17; 18; 20; 23; 24) culture was positive in 55% and images evoking *Fungi* were detected in 65% of the clinical samples by direct microscopic examination (Table 2). In 4 out 10 patients with clinically suspected fungal keratitis and culture negative (samples 29–38), *Fungi* were detected by direct microscopic examination of deep corneal scrapings (filaments in 2; budding yeasts in 1 and pseudohypha in 1) (samples 29; 31; 32 and 36). In addition, for these same 10 culture negative patients, PCR-HRM detected and characterized *Fungi* in 7 out of 10, including the 4 detected by direct microscopic examination. The relative high number of cornea negative cultures could be partially the result of residual eye drop preservatives carried with the samples from the eye surface. PCR-HRM was negative for all the controls carried out with the samples obtained from the air, surfaces, reactants and the blood of healthy donors (total blood or buffy coats) (Table 2).

Table 3 shows the recovery and detection time for haemoculture bottles spiked with *Fung*i. For fungal inoculums containing 100 CFU/bottle, the time for positivity was ranged between 16 and 36 hours of incubation for yeasts and between 36–48 hours for filamentous *Fungi*. Positivity was obtained after 24 to 48 hours of incubation of blood spiked with yeasts and after 24 to 72 hours with filamentous *Fungi*. For inoculums of 10 CFU/bottle the time for positivity was ranged between 24–48 hours for yeasts and between 48 and 72 hours for filamentous *Fungi*. For yeasts suspended in blood cultures were positive after 24–48 hours and after 24–72 h for filamentous *Fungi*. Only *C. albicans*, *Trichosporon* and *Penicillium piccum* could be detected for inoculums containing 1 CFU per bottle (after 96 h of culture) in saline. Inoculums containing 1 CFU per bottle or less of 7 different yeasts or 4 filamentous *Fungi* suspended in blood were negative. PCR-HRM detected 100% of the samples containing the equivalent of 0.1 CFU/ml of yeasts and filamentous *Fungi* and generated reproducible melt-curves. When challenged against profiles obtained from referenced strains run in parallel the melting profiles obtained with all the samples allowed differentiating yeasts from filamentous *Fungi* and discriminating among 7 different strains of yeasts. PCR-HRM performances were equivalent for *Fungi* suspended in saline or blood at concentrations significantly lower (10 times or more) than the detection limits of fungal cultures (Table 3).

## Discussion

The automatic melting analysis of fungal sequences amplified with 2 sets of primers diluted in a mix containing a DNA intercalating dye (SYTO9) allowed rapid detection of *Fungi*. The differences in amplicon sizes between species were suited to fungal differentiation of yeasts from filamentous *Fungi* and to speciation among yeasts. By adapting the existing real-time PCR instrumentation for data acquisition it was possible to carry out reproducible diagnosis in less than 2.30 h after DNA extraction, with detection limits of at least 0.1 CFU of filamentous *Fungi* and yeasts per µl of sample with no need for molecular probes (radioactive, enzymatic or fluorogenic) or post amplification procedures (sequencing, amplicon restriction enzyme analysis, etc.). Corneal samples could be readily assayed after DNA extraction without interference from other DNAs found in the specimens.

Fungal culture performances depend on the type of agent, the fungal load and the mass of material that can be processed, the microbiology laboratory capacities and the presence of antiseptic or antifungal in the samples. Moreover, cultures rely on the ability of the organism to grow *ex vivo* and may require long incubation periods (may become positive late in the course of the infection), are time consuming and prone to contamination. [1], [4], [11].

In 89% of patients with suspected fungal keratitis PCR-HRM was able to detect *Fungi* differentiating filamentous *Fung*i and yeasts. Interestingly, in all patients with positive PCR-HRM the corneal infiltrates were dramatically reduced after antifungal treatments, suggesting that PCR-HRM signals represented true positive infections (results not shown). Local and topical antifungal treatments are long lasting, potentially toxic, and first-line therapies have to selectively target yeasts or filamentous *Fungi*.[4]–[6], [27]–[29] On this matter, yeasts but not filamentous *Fungi* require Fluconazole and/or 5-Fluorocytosine (5FC). *Aspergilli* require Voriconazole and/or Caspofungin; *Candida krusei* are intrinsically resistant to Fluconazole and *Candida glabrata* and *Candida tropicalis* have unpredictable susceptibilities to this latter.[4]–[6], [27]–[29] Hence, false negative diagnosis or lack of discrimination among major fungal types may have a direct impact on infection management (morbidity and mortality).

The yeast detection capacities of the haemoculture system used in the present study are equivalent to those reported for other automated systems (inoculums containing the equivalent of 100 and10 CFU/bottle). Nevertheless, all the blood samples containing fungal loads of 1 CFU/bottle were negative. For automated blood culture bottles inoculated with 1000 yeasts it was shown that the BacT detected growth of 90% of *Candida*, while Bactec 66%. [6], [7] The mean time to growth detection was between 25.62 h and 27.30 h and both detected experimentally infected blood (simulated candidemia) only when additional specialized mycology media were used. [6] For filamentous *Fungi* the BACTEC plus Aerobic/F and the BACTEC Mycosis-IC/F automated systems followed by subculture in solid medium detected inoculums of *A. fumigatus* at concentrations of >3 conidia per 10 ml after 21–40 h, with longer incubation periods for *A. flavus* and *A. terreus*. [7].

For the detection and identification of *Candida* and *Aspergillus* species a variety of real-time PCR assays based on species specific probes were developed targeting the 18 S rDNA [16], [30], the mitochondrial cytochrome *b* [31], the 18 S and 28 S rDNA [32], the CaMP65 (65-kDa mannoprotein) gene [33], the RNase P RNA gene [34], and the ITS 2 region [35]. These nucleic-acid amplification techniques are independent of microorganisms’ growth and detect stressed, injured, and fastidious *Fungi* and those suspended in antimicrobials.[11]–[14] Despite this, expectations for routine direct fungal diagnosis are not fulfilled with PCRs because they a) cover either yeasts or filamentous *Fungi*, b) do not to detect *C. lusitaniae* and c) may require complex post amplification procedures and/or the synthesis and stability testing of labelled molecular probes.[11]–[17], [26] Therefore, DNA sequencing was almost the only tool for molecular species assessment of PCR-positive samples.[36]–[37].

For diagnosis of candidemia in subjects with haematological malignancies or various forms of immunodeficiency a real-time PCR targeting the 18 S rRNA gene and requiring a series of labelled molecular probes, yielded positive results in 58.3% of blood culture-positive samples and detected in blood the genomes of *Candida* 3 days earlier than culture. [37] In this series 27% of whole-blood were PCR positive compared to 15% of haemoculture (92% of correlation of positives). Other studies indicate that numerous pairs of primers and labelled probes were required for each sample to identify 72% of species of positive cultures. [38] For vaginal specimens it was reported a test based on PCR-HRM technology, but it was unable to differentiate yeasts from filamentous *Fungi*. [22] However, the strategy developed here is different because PCR-HRM is able to detect and differentiate yeasts and filamentous *Fungi* in one run with detection limits of 0.1 CFU/µl or less. In addition to differentiating filamentous *Fungi* from yeasts, PCR-HRM discriminated among relevant clinical species of yeasts in less than 2.30 hours after DNA extraction. This only required upgrading the available real-time PCR software of the thermocyclers used in the routine microbiology laboratory.

Because PCR-HRM was able to produce consistent results without the need for synthesizing and labelling molecular probes and without post amplification procedures (restriction enzymes, electrophoresis, gel analysis, hybridisation, sequencing reactants) the cost for reactants for testing one DNA extract could be reduced to less than 2 USD (2 primers and HRM mix). Compared to classic PCR (amplification followed by electrophoreses and/or hybridization and/or sequencing) this new PCR-HRM has the additional advantage of minimizing risks for false positive results due to cross contamination, because the targeted sequence amplification, the signal detection, and the DNA melting analyses are carried out in closed tubes. Moreover, PCR-HRM minimizes risks for false negative results because the yields of extraction of the DNA and the potential interference of PCR inhibitors are systematically monitored in each run and for all the samples. According to the results obtained in this study, if the future runs generate reproducible melt-curves over time in different settings, a reference database could be built to store PCR-HRM calculations and shapes of the melting profiles for each family or species to be challenged against profiles. Larger prospective multicentric trials testing different types of samples (clinical and environmental) are necessary to validate PCR-HRM usefulness as a diagnosis tool and for environmental studies.
